# EID3 directly associates with DNMT3A during transdifferentiation of human umbilical cord mesenchymal stem cells to NPC-like cells

**DOI:** 10.1038/srep40463

**Published:** 2017-01-11

**Authors:** Liang Luo, Wen-Jin Chen, James Q. Yin, Ru-Xiang Xu

**Affiliations:** 1 Bayi Brain Hospital, General Hospital of PLA Army, Southern Medical University, Beijing 100700, P. R. China; 2 Stem Cell Research Center, Neurosurgery Institute of Beijing Military Region, Beijing 100700, P. R. China

## Abstract

There has been recently been increased interest in the plasticity of human umbilical cord mesenchymal stem cells (UMSCs) and their potential in the treatment of neurological disorders. In this study, UMSCs were transdifferentiated into neural stem-like cells (uNSCL), these cells grow in neurosphere-like structures and express high levels of NSCs markers. Epigenetics-related gene screening was here used to assess the relationship between E1A-like inhibitor of differentiation 3 (EID3), a p300 inhibitor, and DNA methyltransferase 3 A (DNMT3A) during the transdifferentiation of UMSCs into uNSCL *in vitro*. Before transdifferentiation of UMSCs into uNSCLs, high levels of EID3 and low levels of DNMT3A were detected; after transdifferentiation, low levels of EID3 and high levels of DNMT3A were detected. The current work showed that EID3 and DNMT3A co-localized in cell nuclei and EID3 interacted directly with DNMT3A in uNSCL. In summary, these results suggest that DNMT3A is probably directly regulated by EID3 during UMSC transdifferentiation into uNSCLs. These findings indicated a novel mechanism by which EID3, a p300 acetyltransferase inhibitor, could directly affect DNMT3A, this enzyme possesses dual methylation and demethylation abilities. These studies may be helpful for understanding a complex regulation mode of DNMT3A, which is a unique member of the methyltransferase family.

Neurodegenerative disorders are great threats to human health, and transplantation of healthy functioning neurons to replace dead neurons is considered to be a viable therapeutic method. However, terminally differentiated neural cells are less likely to survive transplant procedures. Neural progenitor cells (NPCs) can proliferate and subsequently differentiate into almost all neural cell lineages. Therefore, NPCs are a more attractive candidate than fully differentiated neural cells for treating neurodegenerative diseases by transplantation. However, NPCs are limited by the number of brain tissue donors, and other cell sources that can transdifferentiate into neural cells, such as mesenchymal stem cells (MSCs), are potentially ideal alternative cell sources.

MSCs, for example, bone MSCs (BMSCs), adipose MSCs (AMSCs) and umbilical cord MSCs (UMSCs), are sources of multipotent stem cells^1^, and they can transdifferentiate to neural stem-like cells (uNSCL)^2^,^3^,^4^ under natural or experimentally induced conditions^4^,^5^. BMSCs, UMSCs and AMSCs are ideal cell sources for transplantation, since they can transdifferentiate into uNSCL cells under some induced conditions *in vitro* or *in vivo*^6^,^7^. However, the uNSCL are not widely accepted and applied for clinical use, and also may be of very unclear its molecular mechanism. We now know that epigenetic regulation are the key mechanisms during UMSC transdifferentiation into uNSCL. During these processes, MSCs start in a type of pluripotent state, from which they can transdifferentiate into other stem cell types, and progressively develop a different type of pluripotent state, and their gene-expression programmes become huge changes.

Epigenetic gene regulation commonly refers to a heritable process by which regulate gene activity without altering the DNA code. Epigenetics can be described as a bridge between genotype and phenotype, more important, it provides a powerful tool for regulating key of stem cell development features such cell fate determination, commitment, and differentiation. Epigenetic changes of MSC transdifferentiation include chromatin remodeling, histone modification, DNA methylation and microRNA^8^. Thereinto, histone acetyltransferase P300 and DNA methyltransferases Dnmt3a play key roles in histone modification and DNA methylation.

The major function of EID (E1A-like inhibitor of differentiation) gene family is to serve as a p300/CBP suppressor in response to cell transformation, growth arrest or apoptosis. The EID protein family includes EID1^9^, EID2^10^, and EID3^11^. Our research interest EID3 is homologous to a region of EID1, binds to p300/CBP, and acts as an inhibitor of p300/CBP-dependent transcription by direct interactions with nuclear receptors SHP and SRC1^11^.

DNA methylation is also a pivotal mechanism of epigenetic regulation of stem cell reprogramming. Building-up stem cell fates during development depends on de novo DNA methylation catalyzed by DNMT3A and DNMT3B. The function of the DNMT3A protein is very unique: unlike DNMT3B (it defect lead to early embryonic lethality), Dnmt3a-knockout mice get developmental defects and die prematurely^12^, and even more particularly, the methyltransferase exhibits both methylation and demethylation actions, namely, it is involved in CpG methylation and active demethylation of 5mCpGs through deamination^13^, and DNMT3A govern low DNA methylation canyons which span conserved domains frequently containing several essential transcription factors^14^. DNMT3A is also to find a distinct way of self-regulation of inhibition different from the DNMT family other members^15^.

Several recent studies suggest DNMT3A has also a key role in NPC differentiation and central nervous system (CNS) development; DNMT3A is heavily expressed in NPCs, postmitotic CNS neurons, and oligodendrocytes^12^,^16^,^17^,^18^. In studies in mouse embryonic stem cell-derived NPCs, DNMT3A was found to regulate the timing of both differentiation and proliferation. DNMT3A^−/−^ neural stem cells (NSCs) displayed a significant growth in proliferation contrast with wildtype NSCs. For all this, we currently only have few knowledge of DNMT3A’s regulatory mechanism, especially its mode of action of demethylation during cell reprogramming is still obscure.

Using real-time quantitative polymerase chain reaction (qRT-PCR) screening of epigenetics-related genes, we discovered that there is a relationship between *EID3* and *DNMT3A* during the process of UMSC to uNSCL transdifferentiation. Next, we found the protein expression of EID3 is high while DNMT3A is very low in UMSCs; and EID3 is low while DNMT3A is high in uNSCL. Furthermore, EID3 directly interacts with DNMT3A and regulates its expression in uNSCL. This raises the interesting question of whether there is a relationship between EID3 and DNMT3A during uNSCL transdifferentiation. Our study gives new insights into the epigenetic mechanisms of MSC transdifferentiation.

## Results

### Characterization of undifferentiated UMSCs and conversion it into uNSCL

**Figure 1:**
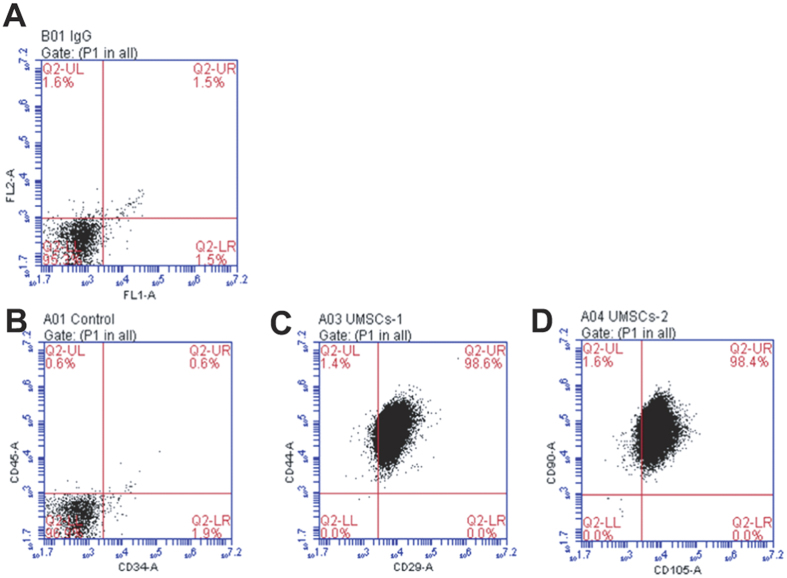
Immunophenotype of human umbilical mesenchymal stem cells. Cell surface markers of human umbilical mesenchymal stem cells (UMSCs) were detected by flow cytometric analysis at passage 3. (**A**) The related isotype control was used as a negative control. UMSCs did not express (**B**) CD34 and CD45, but expressed (**C**) CD29, CD44, (**D**) CD90, and CD105.

**Figure 2:**
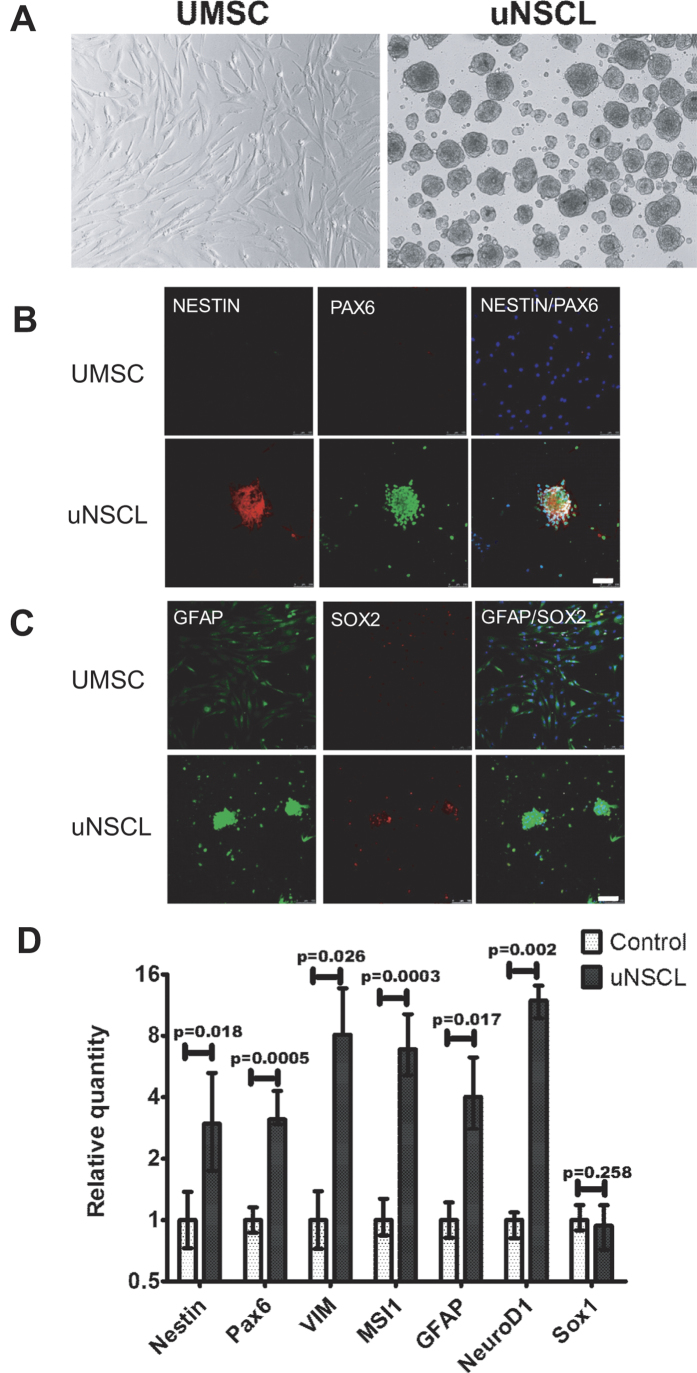
Characteristics of human UMSCs-derived neural stem cell-like cells (uNSCLs) (**A**) Morphology of human UMSCs and uNSCL. (**B**) NESTIN and PAX6 expression of UMSCs (upper panels) and uNSCLs (below panels). (**C**) GFAP and SOX2 expression of UMSCs (upper panels) and uNSCLs (below panels). Scale bars represent 100 μm. (**D**) Quantitative real-time RT-PCR analyses of NSC marker gene expression (*Nestin, Pax6, VIM, MSI1, GFAP, NeuroD1, SOX1*). Expression levels are expressed relative to the housekeeping gene *GAPDH*. Data were analyzed by one-way ANOVA, the experiments were performed at least three times. Abbreviations: UMSCs, umbilical cord mesenchymal stromal cells; uNSCL, UMSCs-derived neural stem cell-like cells; GAPDH, Glyceraldehyde 3-phosphate dehydrogenase.

Flow cytometry showed that UMSC were CD29^+^, CD44^+^, CD105^+^, CD90^+^, CD34^–^, CD45^–^(Fig. 1)^19^,^20^,^21^. To convert hUMSCs into cells with characteristics of NSCs, we detached UMSCs after 4–6 passages and cultured them in serum-low medium (2–3% FBS) supplemented with EGF and bFGF (see Methods for details). uNSCL proliferated with an estimated doubling time of 2.6 days *in vitro* for at least up to 8 weeks without visibly changing morphology or phenotype (Fig. 2A).

Immunocytochemistry showed that uNSCL expressed high levels of Nestin, GFAP and Pax6, and some cells expressed Sox2 (Fig. 2B,C). Quantitative RT-PCR of uNSCL, mRNA encoding *NESTIN, PAX6, VIMENTIN, GFAP, MUSASHI1* and *NEUROD1* could be detected at levels between 3- and 13.2-fold those seen in UMSCs (*P *= 0.018, 0.005, 0.26, 0.0003, 0.017, and 0.002, respectively) (Fig. 2D).

### EID3 and DNMT3A expression patterns in uNSCL, UMSCs and NSCs

**Figure 3:**
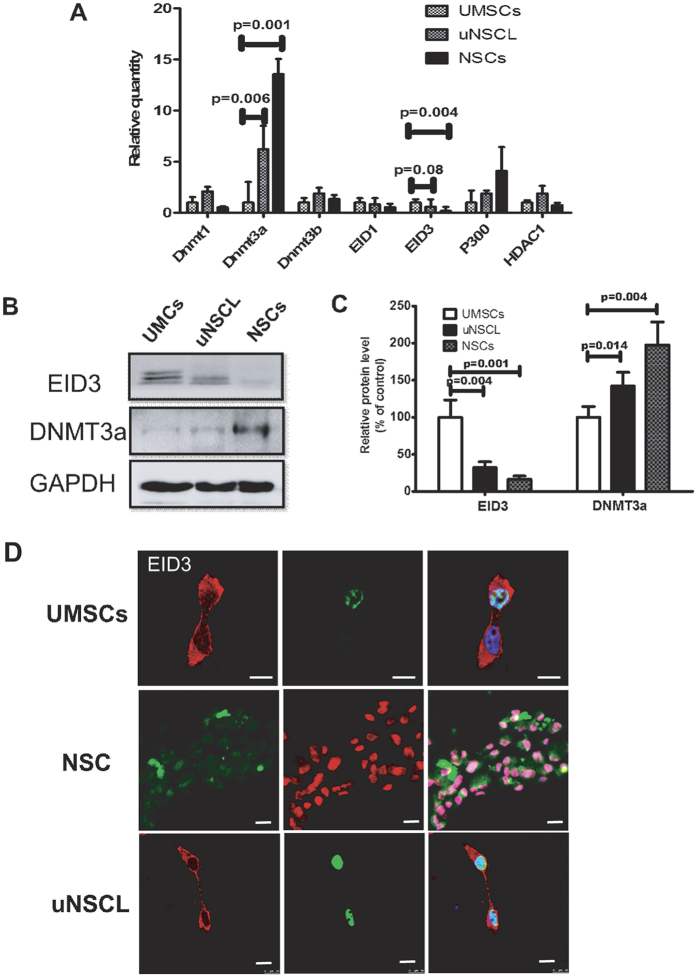
The expression of EID3 and DNMT3A in UMSC, uNSCL and NSC cells. (**A**) Screen of relative epigenetic gene expression (*DNMT1, DNMT3A, DNMT3B, EID1, EID3, p300, HDAC1*) by qRT-PCR. Expression levels are expressed relative to the housekeeping gene GAPDH. (**B**) Western blotting protein analysis of EID3 and DNMT3A in UMSCs, uNSCL and NSCs. GAPDH expression was used as a protein loading control. (**C**) Histogram of the densitometric quantification of data presented in the left panel. Data were analyzed by one-way ANOVA, the experiments were performed at least three times. (**D**) Intracellular localization of EID3 and DNMT3A; EID3 and DNMT3A were expressed in UMSCs, uNSCL, and NSCs as detected by immunofluorescence using EID3 (green) and DNMT3A (red) antibodies. Nuclei were stained with DAPI (blue). More than 30 cells were studied. Scale bars represent 10 μm. Abbreviations: DNMT 3 A/3B, DNA methyltransferases 3 A/3B; EID3, E1A-like inhibitor of differentiation 3; p300, E1A binding protein p300; HDAC1, Histone Deacetylase 1; UMSCs, umbilical cord mesenchymal stromal cells; uNSCL, UMSCs-derived neural stem cell-like cells; GAPDH, Glyceraldehyde 3-phosphate dehydrogenase.

To investigate the relationship between methylation and acetylation enzymes in uNSCL cells, we analyzed the expression of several epigenetics-related genes, including *DNMT1, DNMT3A, DNMT3B, HDAC1, P300, EID1,* and *EID3* by qRT-PCR (Fig. 3A). Results showed that the expression level of Dnmt3a expression was significantly increased in uNSCL (*P* = 0.006) and NSC (*P* = 0.001) cells relative to UMSCs (Fig. 3A).

Next, we examined the protein expression level of DNMT3A and EID3 in UMSC, uNSCL and NSC cells, as shown in Fig. 3B. DNMT3A expression levels varied across the cell types: in UMSCs, the expression level of EID3 was higher than that of DNMT3A, whereas, in NSCs, the opposite was the case. It is interesting that EID3 and DNMT3A were both detected in uNSCL cells, and their protein expression levels were detected in UMSCs and NSCs (Fig. 3B,C).

### EID3 and DNMT3A co-localize in uNSCL cells

Previous studies have demonstrated that EID3 interacts with p300/CBP^11^, and both DNMT3A and CBP were shown to interact with PU.1^22^,^23^. This suggests that there may be a mechanistic link between EID3 and DNMT3A. To confirm this hypothesis and obtain further insight into the interactions between DNMT3A and EID3, we investigated their cellular localization by immunocytochemistry and confocal microscopy.

An analysis of sub-cellular localization determined that EID3 and DNMT3A preferentially accumulate in the cell nucleus. EID3 was expressed in both the cytoplasm and nucleus of cells (Fig. 3D), and the majority of DNMT3A was discovered in cell nuclei and co-localized strongly with EID3 (Fig. 3D). These results support the hypothesis that DNMT3A interacts with elements of EID3.

### EID3 directly interact with DNMT3A

**Figure 4:**
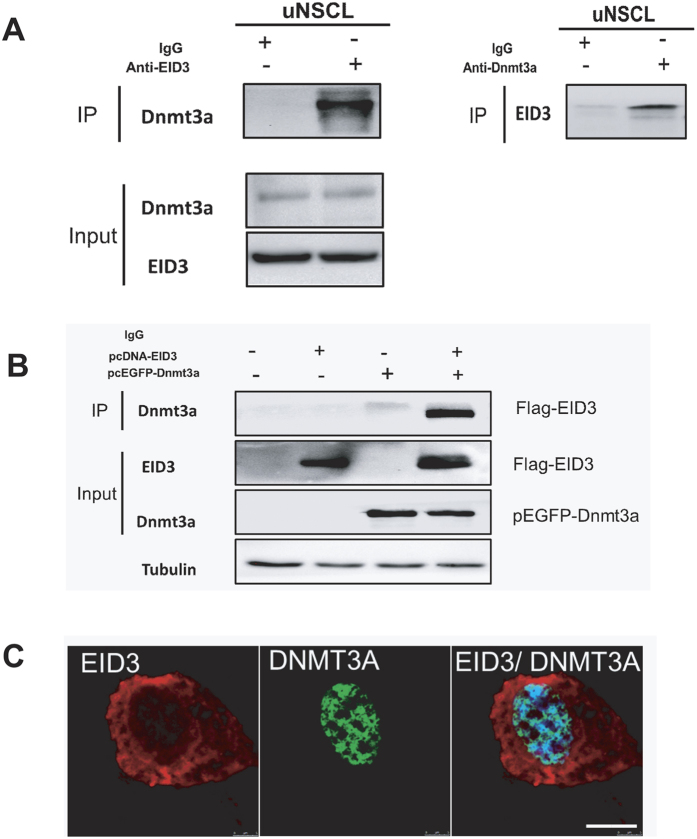
EID3 interacts with DNMT3A. (**A**)EID3 co-immunoprecipitates with endogenous DNMT3A in uNSCL. Endogenous EID3 is associated with DNMT3A. (**B**) EID3 co-immunoprecipitates with DNMT3A in HEK293 cells. pcDNA3.1-EID3, pEGFP-DNMT3A, or their control vectors were co-transfected into HEK293 cells, cells were harvested and lysed with Co-IP lysis buffer after 48 h transfection. Cells lysates were incubated with anti-EID3 antibody and protein A/G beads at 4 °C overnight followed by immunoblotting. (**C**) EID3 and DNMT3A were expressed in HEK293. Scale bars represent 10 μm.

Endogenous EID3 is associated with the DNMT3A complex in uNSCL. The immunocytochemistry results showed that EID3 likely interacts with DNMT3A. Next, we further examined the possibility that EID3 interacts with DNMT3A in uNSCL cells by co-IP analysis. Results showed that anti-EID3 antibody could precipitate endogenous DNMT3A, meanwhile anti-DNMT3A also precipitate endogenous EID3 (Fig. 4A).

Exogenous EID3 and DNMT3A interact in HEK293 cells. We cloned EID3 and DNMT3A and found that recombinant Flag-EID3 protein could precipitate recombinant pEGFP-DNMT3A. This association was verified in co-transfected HEK293 cells by Co-IP, which also indicates a direct interaction between EID3 and DNMT3A (Fig. 4B).

## Discussion

Since mature nerve cells have a limited capacity for self-renewal, NSCs play an important role in cell therapy for the treatment of neurodegenerative disorders. The question of how to quickly obtain a large number of NSCs is a significant issue in regenerative medicine. Fortunately, MSCs offer a possible source of NSCs, since they are relatively abundant, are easy to isolate, proliferate quickly, and reports indicate that they can be induced to transdifferentiate to a large number NSC-like cells in a short period^4^,^24^. However, there has been controversy over whether MSCs can naturally transdifferentiate to NSCLs and not just through induction with exogenous transcription factors.

In our studies, uNSCL, which were transdifferentiated from UMSCs using the sphere culture method^4^, could be passaged for at least six generations. Nonetheless, we believe that uNSCL represent an intermediate state of which MSCs was transdifferentiated to NSCs, mainly for the following reasons: 1) In serum-free culture media, UMSCs are more prone to aggregation and formed spheres, so the spheres are not fully equal to NSC/NPC neurospheres which formed neurospheres reasons mainly due to cell proliferation; 2) although uNSCL cells expressed many surface and transcription factor markers of NSCs, but some important NSC markers, such as SOX2, were not increased significantly. Despite these issues, uNSCL cells can proliferate by forming spheres, express relevant markers of NSCs, and differentiate into different neural cell lineages. Therefore, uNSCL cells should be valuable to be a model to study MSC transdifferentiation NSCs.

In somatic cell reprogramming, the balance of DNA methylation and demethylation will maintain the DNA methylation profiles, but the demethylation mechanism is still unclear. We now know that a *de novo* methyltransferase DNMT3A still has dehydroxymethylase activity^25^, several studies have reported that the demethylation process is initiated by DNMT3A^13^,^26^. However, during cells transdifferentiation, how DNMT3A is adjusted to adapt methylation or demethylation role is still unknown. We measured EID3 and DNMT3A expression in three cell states uNSCL, UMSCs, and NSCs, and DNMT3A and EID3 were found to have a relationship, then we confirmed EID3 directly interact with DNMT3A during UMSCs transdifferentiation. These results imply EID3 may participate in regulation of methylation or demethylation process of DNMT3A and affect the balance of demethylation and methylation during UMSCs transdifferentiation to uNSCL.

There has a relationship between a P300 inhibitor EID3 and methyltransferase DNMT3A may reflect complex epigenetic regulation during MSCs transdifferentiation, indicating the need to take multiple factors into account as one seeks to understand transdifferentiation mechanism.

## Materials and Methods

### Ethical approval

In this study, no vertebrate animals or human subjects were used, and all of the experiments are carried out at the cellular and sub-tissue (part of the umbilical cord) level. All human experiments performed throughout the present study were approved by the Human and Animal Research Ethics Committees of General Hospital of PLA Army.

All relevant experiments were carried out in accordance with the approved regulations and guidelines by the Ethics Committees according to the Regulation on Ethical Review of Biomedical Research Involving Human Subjects promulgated by MOH of China.

### Isolation of human UMSCs

All clinical procedures followed the protocols approved by the Human and Animal Research Ethics Committees of General Hospital of PLA Army. Human umbilical cord collected from consenting mother for the current study, and written informed consent was obtained from every donor. Isolation of human UMSCs was performed as previously described with slight modified^19^. In brief, fresh human umbilical cords were obtained after birth and following disinfection in 75% ethanol for 30 s and stored in Hanks’ balanced salt solution for 1–6 hours before tissue processing to obtain mesenchymal cells. After the arteries and veins were removed, and the tissue was move to a container in DMEM/F12 and cut into 2–4 mm^3^ pieces fragments, and incubated with an enzyme solution (comprised 0.5 mg/mL of collagenase, 0.5% trypsin and 0.5 mg/mL of hyaluronidase) for 45 to 60 min at 37 °C. Then, tissue was crushed with forceps to release individual UMSC cells, and large pieces of tissue were removed. The cells were pelleted by 250g for 5 minutes centrifugation, suspended in fresh growth medium (containing the DMEM/F12 supplemented with, 10% FBS and 1% penicillin-streptomycin).

### Culturing Cells

Isolated UMSCs were cultured in DMEM/F12 supplemented with 10% fetal bovine serum (FBS) and glucose (4.5 g/l) at 37 °C with 5% CO2 at saturating humidity. When cells reached about 80% confluency, the cells were detached with 0.25% trypsin-EDTA (Life Technologies, Ltd), the trypsin was inactivated with serum-containing media. The cells were cultured for 2–4 passages before use.

HEK293 were cultured in DMEM supplemented with 10% FBS, 2 mM glutamine, penicillin (100 U/ml), and streptomycin (0.1 mg/ml). Cultures were kept at 37 °C containing 5% CO2 in a humidified incubator.

Human NSCs (Millipore, SCC007) were routinely expanded according to the manufacturer’s protocol. The NSCs were maintained in laminin coated culture dishes precoated with poly-L-lysine in ReNCell media (Millipore) supplemented with basic fibroblast growth factor (bFGF, 20 ng/ml, Peprotech) and epidermal growth factor (EGF, 20 ng/ml, Peprotech). The human NSCs were plated in the dishes at a cell density of 5 × 10^5^/ml. All NSC experiments were carried out between passages of 3 and 10.

### Generation of uNSCL from UMSCs

We referred previous method to obtain uNSCL (neural progenitor cell-like cells neurospheres) from UMSCs (umbilical cord mesenchymal cells) were performed as previously described^4^,^27^ with minor modifications. In brief, UMSCs were dissociated with 0.05% trypsin and plated on low-attachment plastic tissue culture plates (Corning Inc., Lowell, MA, USA) at a concentration of 1.5–2 × 10^5^ cells/cm^2^ in NSC culture medium containing the N2B27 medium supplemented with 20 ng/mL of both epidermal growth factor (EGF) and basic fibroblast growth factor (bFGF). On the third or four day after plating, cell clusters were collected by centrifugation, dissociated with Accutase (Sigma, USA), and replated on low-attachment plastic tissue culture plates at a concentration of 1.5–2 × 10^5 ^cells/cm^2^ in the NSC culture medium. The neurospheres were apparent within 7–10 days in the culture and ready for related experiments.

### Flow cytometry

UMSCs were treated with trypsin-EDTA and washed twice with PBS containing 1% FCS and 0.02% sodium azide. Dead cells were excluded from analysis by forward-scatter gating. Samples were analyzed using FACSCalibur flow cytometer and software (Becton Dickinson, Franklin Lakes, NJ). A minimum of 20,000 events was acquired for each sample.

### Plasmids

Human EID3 and DNMT3A of Full-length cDNAs was amplified by PCR from human brain cDNA library. The pcDNA3.1-EID3 and pEGFP3-Dnmt3a were generated by subcloning the corresponding DNMT3A cDNA into pEGFP-C1 (Clonetech) and EID3 cDNA into pcDNA3.1 (Invitrogen) vector, respectively. Plasmid DNA was purified on Qiagen midi prep columns and sterilized using a 0.22μM filter. Superhelicity of DNA for transfection experiments was determined by electrophoresis on 0.7% agarose–ethidium bromide gels. Only highly supercoiled (>90%) preparations of DNA were used for transfection. All constructs were confirmed by sequencing.

### Antibodies

The following antibodies were used for Western blotting, immunoblotting and Flow cytometry: Dnmt3b (Abcam Inc., Cambridge, MA); DNMT3A (Cell Signaling Technology, Beverly, MA); beta-Actin (Abcam Inc., Cambridge, MA); EID3 (Abcam Inc., Cambridge, MA); CBP/p300, 1:1000 (Cell Signaling Technology, Beverly, MA); GAPDH (Abcam Inc., Cambridge, MA); FITC-CD34, FITC-CD29, FITC-CD106, PE-CD45, PE-CD44, PE-CD90 (BD Biosciences, Piscataway, NJ, USA).

### Western blotting

Total proteins were extracted using the RIPA lysis buffer supplemented with the protease inhibitor cocktail (Thermo Fischer, Pittsburgh, PA). Protein concentrations were determined by a BCA Protein Assay kit (Beyotime, China). Equal amounts of proteins were separated by SDS-PAGE, and transferred onto the PVDF membranes (0.45 μm pore size, Millipore, USA). Blots were incubated with the 5% BSA for 1 hr at room temperature, and probed overnight at 4 °C with the corresponding antibodies. On the next day, membranes were thoroughly rinsed in the phosphate-buffer saline (PBS) containing 0.1% Tween-20 (PBST), and incubated with horseradish peroxidase-conjugated goat anti-rabbit IgG or goat anti-mouse IgG secondary antibodies for 1 hr. Bands were then visualized by the ECL detection kit (GE Healthcare Life Science, Pittsburgh, PA), and documented on films. Intensities of bands were analyzed by densitometry using the Quantity One software (Bio-Rad, Hercules, CA).

### Co-immunoprecipitation assay

Cell extracts were prepared in ice-cold RIPA buffer (50 mM Tris-HCl, 150 mM NaCl, 0.5% sodium desoxycholate, 0.1% SDS, 5 mM EDTA, 2 mM PMSF, 20 mg/mL aprotinin, 20 mg/mL leupeptin, 10 mg/mL pepstatin A, 150 mM benzamidine, and 1% Nonidet P-40) for 30 minutes. After protein A/G beads (Santa Cruz, CA, USA) were washed with dilution buffer for 2 h at 4 °C, lysates were used for immunoprecipitation with the indicated antibodies.

### Immunocytochemistry and confocal microscopy

Cells were seeded as 1.5 × 10^4^ cells/cm^2^ on cover slips in 12-well plates, and cells were fixed in 4% paraformaldehyde in phosphate-buffered saline (PBS). Permeabilization and blocking were performed in 5% BSA and 0.25% Triton X-100 in PBS for 30 min. Cells were stained with primary antibody at 4 °C overnight. Secondary antibody was applied for 2 h at room temperature. Images were acquired using a Leica confocol and software. Sub-cellular images were determined using a confocal imaging system (CLSM, TCS SP5 II, Leica).

Cells were fixed in 4% paraformaldehyde in PBS. Immunocytochemistry was carried out using standard protocols. Fluorescence labeled secondary antibodies (donkey anti-rabbit secondary antibody Alexa Fluor 488, donkey anti-mouse IgG Alexa Fluor 568) were then applied. Cell nuclei were counterstained with 4,6-diamidino-2-phenylindole (DAPI).

### RNA extraction, RT-PCR and quantitative real-time RT-PCR analysis

RNA was isolated from cells using TRIzol (Invitrogen) according to the manufacturer’s protocol. Total cellular RNA was extracted from UMSCs, NSCs and uNSCLs using TRIzol (Invitrogen) followed by treatment with RNase-free DNase according to the manufacturer’s protocol.

To determine the expression levels of mRNA, total RNA was reverse transcribed with a PrimeScript® RT Reagent Kit (TaKaRa, Otsu, Japan). Approximately 500 ng of total RNA was used for the first strand cDNA synthesis. Quantitative real-time RT-PCR was carried out using Applied Biosystems® ViiA™ 7 System, and subsequently amplified using SYBR Green PCR Master mix (TaKaRa, Otsu, Japan) and 0.5 μM each of the sense and antisense primers. After amplification, melting curves of the RT-PCR products were acquired to demonstrate product specificity. Results are expressed relative to the housekeeping gene GAPDH. Primer sequences, lengths of the amplified products are summarized in Table s1.

### Statistical analysis

The SPSS statistical software package (Chicago, IL) was used for statistical analysis. All experiments were performed at least in triplicates. Data were presented as mean± standard deviation (SD). Comparisons were accomplished by one-way analysis of variance (ANOVA) with LSD post-hoc test or t-test. A statistical significance was defined as *P *< 0.05.

## Additional Information

**How to cite this article**: Luo, L. *et al*. EID3 directly associates with DNMT3A during transdifferentiation of human umbilical cord mesenchymal stem cells to NPC-like cells. *Sci. Rep.* **7**, 40463; doi: 10.1038/srep40463 (2017).

**Publisher's note:** Springer Nature remains neutral with regard to jurisdictional claims in published maps and institutional affiliations.

## Supplementary Material

Supplementary Dataset 1
