# Supplementary material for: EID3 directly associates with DNMT3A during transdifferentiation of human umbilical cord mesenchymal stem cells to NPC-like cells
Source: Sci Rep. 2017 Jan 11;7:40463. doi: 10.1038/srep40463 (PMC5225425; doi:10.1038/srep40463)
Supplement: Supplementary Dataset 1 [file srep40463-s1.doc]

**Supplementary material for:**

EID3 directly associates with DNMT3A during transdifferentiation of human umbilical cord mesenchymal stem cells to NPC-like cells

Liang Luo^1,2#^, Wen-Jing Chen^1,2#^, James Q. Yin^1,2*^, Ru-Xiang Xu^1,2*^
*^1^**Stem Cell Research Center, Neurosurgery Institute of Beijing Military Region, Beijing 100700, PR China*

*^2^Bayi Brain Hospital, General Hospital of PLA Army, Southern Medical University, Beijing 100700, PR China*

Key words：umbilical cord mesenchymal stem cells; neural stem cells; reprogrammed; neural progenitors cells; NSCs;

# These two authors contributed equally to this work.

*Correspondence and requests for materials should be addressed Q.Y. (email:clayluo@126.com) or R.X. (email:DrXuruxiang@126.com)

**Table s1. Primer sets for PCR reactions**

|  | **Forward (5' to 3')** | **Reverse (5' to 3')** |
| --- | --- | --- |
| hGAPDH | AGCCACATCGCTCAGACACC | TGAGGCTGTTGTCATACTTCTC |
| hSox2 | CAAAAATGGCCATGCAGGTT | AGTTGGGATCGAACAAAAGCTATT |
| hPax6 | GTCCATCTTTGCTTGGGAAA | TAGCCAGGTTGCGAAGAACT |
| hNestin | GAAACAGCCATAGAGGGCAAA | TGGTTTTCCAGAGTCTTCAGTGA |
| hDnmt3a | GACAAGAATGCCACCAAAGC | CGTCTCCGAACCACATGAC |
| hDnmt3b | GCCGTTCTTCTGGATGTTTGAG | ATCCTATTGTATTCCAAGCAGTCC |
| hEID1 | CTCAGTGGTGCCGGCTACA | CAGGGCTGGTTCTCTTGTTCTC |
| hEID3 | GCCGACGTAGACCCAAAGC | GTTAAGGAGTTGTTCGCCGAG |
| hP300 | GGGACTAACCAATGGTGGTG | ATTGGGAGAAGTCAAGCCTG |
| hHDAC1 | CAAGCTCCACATCAGTCCTTCC | TGCGGCAGCATTCTAAGGTT |
